# Proflavine (PFH^+^): as a photosensitizer (PS) biocatalyst for the visible-light-induced synthesis of pyrano [2,3*-d*] pyrimidine scaffolds

**DOI:** 10.3389/fchem.2024.1304850

**Published:** 2024-03-26

**Authors:** Farzaneh Mohamadpour, Ali Mohammad Amani

**Affiliations:** Department of Medical Nanotechnology, School of Advanced Medical Sciences and Technologies, Shiraz University of Medical Sciences, Shiraz, Iran

**Keywords:** proflavine (PFH^+^), photosensitizer (PS) biocatalyst, photoinduced-electron transfer (PET), renewable energy source, aqueous media, pyrano [2, 3-d] pyrimidine scaffolds

## Abstract

A sustainable methodology for the synthesis of pyrano [2,3-*d*] pyrimidine scaffolds have been developed, employing the Knoevenagel-Michael tandem cyclocondensation reaction of barbituric acid/1,3-dimethylbarbituric acid, malononitrile, and aryl aldehydes. This study elucidates the advancement of a sustainable and environmentally conscious approach to synthesizing this category of chemical compounds. In the present investigation, a novel photosensitizer comprising proflavine (PFH^+^) bio-photocatalyst was employed in an aqueous medium, subjected to air atmosphere at room temperature, and stimulated by a blue-light-emitting diode (LED) to harness renewable energy. The fundamental objective of this initiative is to utilize a photosensitizer (PS) biocatalyst that has been recently developed, can be conveniently acquired, and is priced affordably. The proflavine (PFH^+^) photocatalyst, demonstrates the ability to initiate photoinduced-electron transfer (PET) through exposure to visible light. This property endows the photocatalyst with a practical and efficient method of achieving high effectiveness, energy efficiency, and environmentally friendly outcomes. The current research endeavor has the objective of examining the turnover number (TON) and turnover frequency (TOF) pertaining to pyrano [2,3-*d*] pyrimidine scaffolds. Moreover, it has been validated that cyclization at the gram-scale is a feasible approach that can be employed in various industrial settings.

## Introduction

The utilization of photoredox catalysis offers discernible merits towards sustainability, whilst simultaneously adhering to various tenets of green chemistry. The principal source of energy for light is radiation. Photonic radiation is characterized by its absence of charge, non-toxicity, and ecological compatibility, which renders it an efficient source of energy. The utilization of photons, due to their ability to furnish adequate energy, is a viable approach to attain the coveted reactivity, without resorting to the employment of extreme temperatures or rigorous conditions associated with thermal activation. The utilization of light-absorbing species, also known as photocatalysts, is a viable option for catalysis at low concentrations. The implementation of catalysis at low concentrations is known as the use of catalytic amounts. The term “reagents” refers to the chemical substances used in a reaction to cause a chemical change in the target molecule. By attaining an electronically excited state, they elicit occurrences of electron transfer (ET) either towards or away from inactive/stable substrates. The aforementioned phenomenon engenders notably active entities in a moderate and regulated fashion. There are two favorable outcomes in terms of sustainability that ensue from this situation. Initially, a plausible means of reducing reactivity involves employing low-energy reagents, thereby promoting less dangerous synthetic pathways and facilitating the elimination of less toxic or pollutant byproducts. Photoredox catalysis has the capability to activate typically unreactive groups within molecules. This study demonstrates the enhanced capacity for functional group tolerance exhibited by a particular chemical reaction that involves the cleavage of C-H bonds. Photoredox catalysis is an indispensable tool in the construction of concise synthetic routes with improved atom economies that utilize sustainable feedstock resources (Crisenza and Melchiorre, 2020; Mohamadpour, 2022a; Mohamadpour, 2022b; Mohamadpour, 2023a; Mohamadpour, 2023b).

Proflavine derivatives have garnered significant interest as chemotherapeutic agents owing to their diverse biological activities, which offer promising pharmaceutical potential (Sabolova et al., 2020). Proflavine, which was among the first identified antibacterial agents, was subsequently surpassed by the discovery of penicillin in the 20th century. The utilization of the agent persists in contemporary times as a potent disinfectant (Acheson, 1973). Extensive research on proflavine derivatives has resulted in their prevalent use as agents for antibacterial, anticancer, antimalarial, and antiprotozoal purposes (Denny et al., 1994; Denny, 2002). The significant function of flavins as essential photoreceptors and redox cofactors in various biological processes has subsequently fueled interest in the utilization of flavin derivatives as biomimetic photocatalysts (Ghisla and Massey, 1989; Silva and Edwards, 2006).

The photosensitizer (PS) is a fundamental component involved in the primary processes of light absorption and electron transfer in photocatalytic systems. According to prior reports, a majority of photocatalytic systems employ complexes containing precious metals such as Ru or Ir as a photosensitizer (PS). Unfortunately, the high cost associated with such precious metals has hindered their potential applications (Meng et al., 2021). Proflavine is identified as one of the flavine derivatives. The compound known as proflavine possesses a high degree of photosensitivity, thereby rendering it a subject of significant research interest. In particular, much attention has been devoted to the PFH^+^ strain of proflavine due to its remarkable photophysical and photochemical characteristics. In the present study, a cost-effective organic dye, namely proflavine (PFH^+^), characterized by its affordable pricing and relatively high photo absorption capacity (Meng et al., 2021; Ong et al., 2022), has been chosen to facilitate a facile synthesis of heterocyclic compounds through a sustainable and environmentally conscious route.

The considerable reserves of energy, cost-effectiveness along the capacity to access sustainable energy sources render visible light irradiation a dependable means for the development of organic compounds (Mohamadpour, 2021a; Mohamadpour, 2021b; Mohamadpour, 2022c).

It is anticipated that pyranopyrimidines will exhibit compelling pharmacological and biochemical properties, including inhibitor of the antiallergic (Kitamura and Onishi, 1984), antihypertensive (Furuya and Ohtaki, 1994), cardiotonic (Heber et al., 1993), bronchiodilator (Coates, 1990), antibronchitic (Sakuma et al., 1991), and antitumor activities (Broom et al., 1976).

Numerous catalysts have demonstrated the ability to generate synthetic pyrano [2,3-*d*] pyrimidine scaffolds, for instance, DABCO-based ionic liquids (Seyyedi et al., 2016), L-proline (Bararjanian et al., 2009), iron ore pellet (Sheihhosseini et al., 2016), nano-sawdust-OSO_3_H (Sadeghi et al., 2015), Al-HMS-20 (Sabour et al., 2015), TSA/B(OH)_3_ (Khazaei et al., 2015a), Mn/ZrO_2_ (Maddila et al., 2015), cellulose-based nanocomposite (Maleki et al., 2017), DBA (Bhat et al., 2016), TBAB (Mobinikhaledi and Bodaghi Fard, 2010), Fe_3_O_4_@SiO_2_@(CH_2_)_3_-Urea-SO_3_H/HCl (Zolfigol et al., 2016), Et_3_N-Ultrasonic (Azarifar et al., 2012), ZnFe_2_O_4_ nanoparticles (Khazaei et al., 2015b), microwave (Devi et al., 2003), nickel nanoparticles (Khurana and Vij, 2013), CaHPO_4_ (Bodaghifard et al., 2016), Zn [(L)proline]_2_ (Heravi et al., 2010), theophylline (Mohamadpour, 2021c), β-CD (Mohamadpour, 2022d), and CuO/ZnO nanocatalyst (Albadi et al., 2015). Numerous determinants, including extended reaction periods, utilization of expensive compounds, intricate chemical reactions, and reduced yields, have noteworthy implications for the management and disposal procedures of waste products. Additionally, the process of extracting homogeneous catalysts from reaction mixtures can pose a significant difficulty. Recently, the use of visible-light-induced photochemical reactions has attracted the attention of researchers and many advances have been made so far (Ma C. H. et al., 2022; Ma C. et al., 2022; Danfeng et al., 2022; Mohamadpour, 2022e; Mohamadpour, 2023c). The current investigation delineates the use of photocatalysts in the synthesis of heterocyclic compounds, with a particular emphasis on the incorporation of eco-friendly methodologies. Through the examination that was conducted, it was discovered that organic dye photo-redox catalysts can be easily obtained and are also economically viable. The technique discussed above entails the utilization of a photosensitizer (PS) biocatalyst as a highly effective organo-photocatalyst.

The present investigation has successfully discerned a new type of photosensitizer (PS) biocatalyst, denoted as proflavine (PFH^+^) as a photocatalyst that functions through a photoinduced-electron transfer (PET) pathway induced by visible light. The present protocol employs the Knoevenagel-Michael cyclocondensation reaction, which involves the application of barbituric acid/1,3-dimethylbarbituric, malononitrile, and aryl aldehydes. Moreover, the aforementioned process has the potential to utilize a blue-light-emitting-diode (LED) as a sustainable and environmentally conscious means of renewable sourcing energy in an air atmosphere at room temperature, within a water-based environment.

## Experimental

### General

The melting points of the diverse compounds were ascertained utilizing an electrothermal apparatus denoted as the 9100. The acquisition of ^1^HNMR spectra using DMSO-d_6_ was accomplished through the usage of the Bruker DRX-300 Avance instruments. The aforementioned compounds were generously supplied in significant amounts by Fluka, Merck, and Acros, and were promptly utilized.

### The present study introduces a methodology for the sustainable synthesis of pyrano [2,3-d] pyrimidines with eco-friendly attributes (4a-t)

A solution consisting of 3 mL of water and 0.2 mol% of proflavine (PFH^+^) was prepared at room temperature. The mixture was afterward amalgamated with 1,3-dimethylbarbituric acid/barbituric acid (3, 1.0 mmol), malononitrile (2, 1.0 mmol), and aryl aldehydes (1, 1.0 mmol). The reactions were recorded utilizing thin-layer chromatography (TLC). After the chemical reaction, the crude solid underwent a screening process and was subsequently washed with water. The resulting product was then subjected to crystallization using ethanol, which effectively eliminated the need for any additional purification techniques. The current investigation concerns the possibility of synthesizing the aforementioned substances on a gram-scale, utilizing the domain of pharmaceutical research and development (R&D). A singular experimental procedure entailed the utilization of a combination consisting of 2,4-dimethoxybenzaldehyde, malononitrile, and barbituric acid at a molar quantity of 50 mmol. After a reaction time of 6 min, the resultant product was recovered through the use of a conventional filtration technique. According to the ^1^HNMR spectroscopy data, the chemical compound under investigation demonstrates a substantial level of spectroscopic purity. The spectroscopic data are presented in the Supplementary Material.

## Results and discussion

**FIGURE 1:**
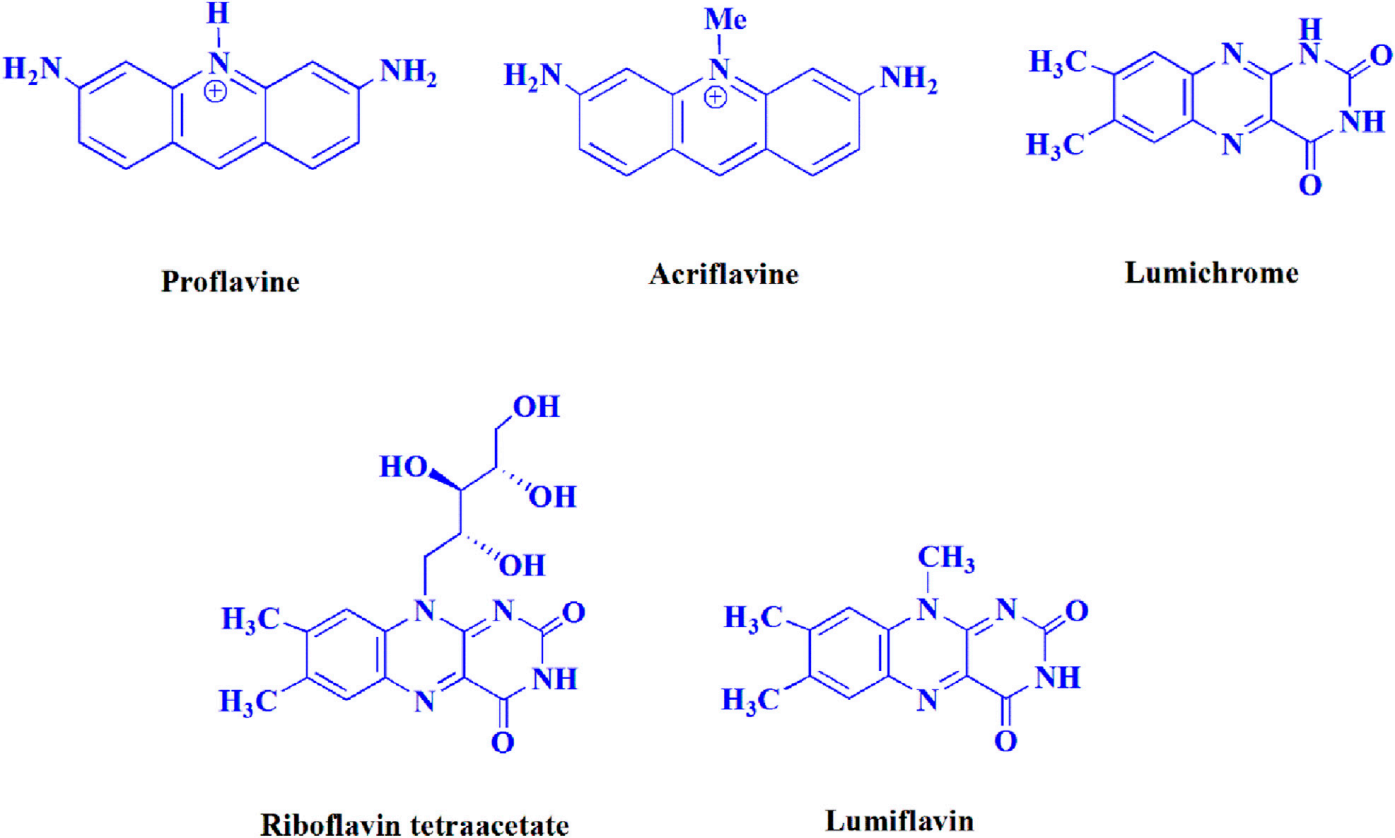
In the present investigation, the effectiveness of the catalyst was evaluated.

**SCHEME 1:**
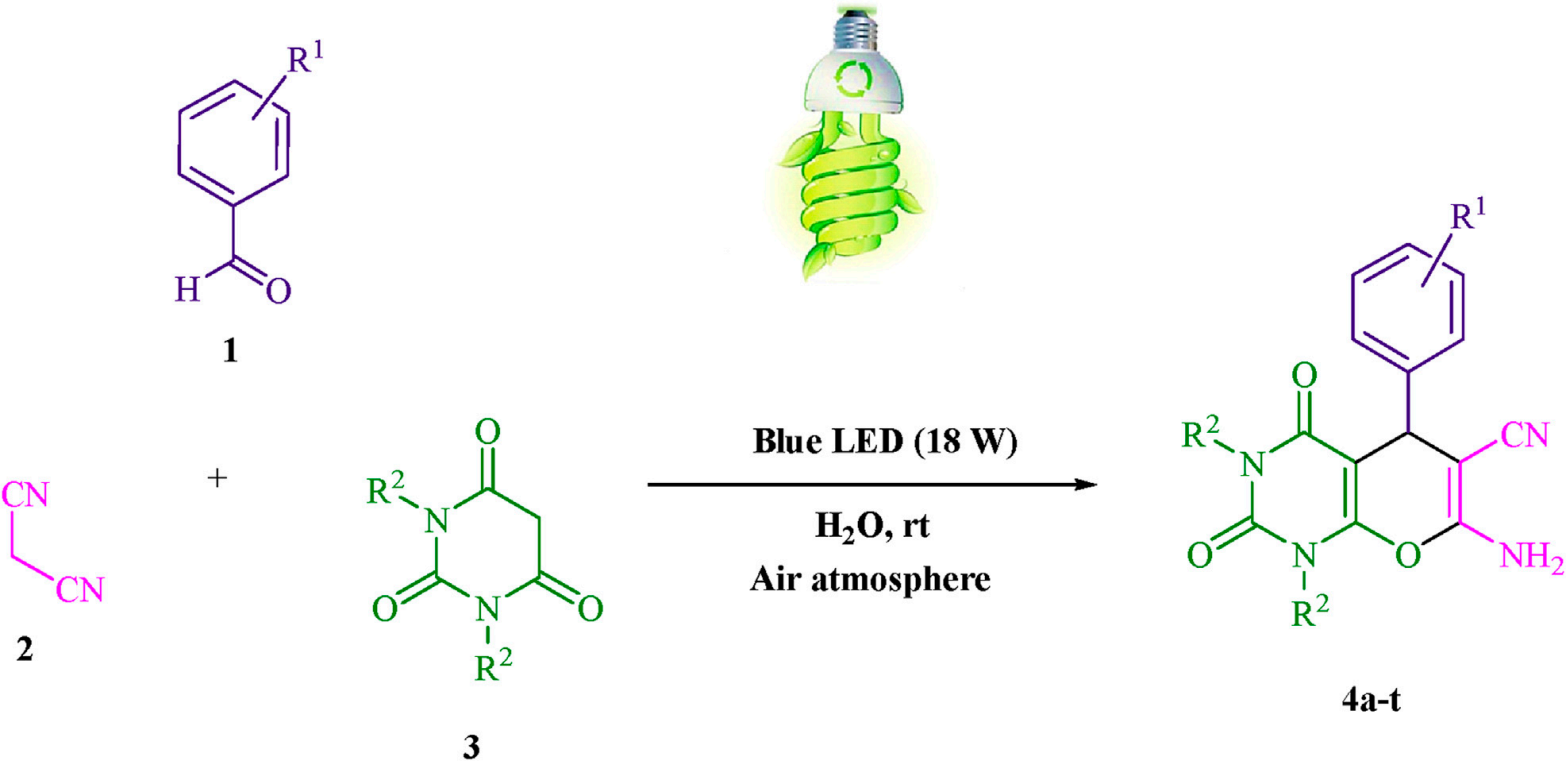
The current study presents a methodology for the synthesis of pyrano [2,3-*d*] pyrimidine scaffolds in a radical manner.

The current investigation ascertained the response of benzaldehyde, malononitrile, and barbituric acid in a 3 mL aqueous solution. Under laboratory conditions, a 15-min incubation of 3 mL of water without the implementation of a photocatalyst resulted in the generation of 4i, which accounted for a proportion of 36%, at ambient temperature. Table 1, entry 8 provides a comprehensive record of the aforementioned observation. The addition of multiple adjunct photocatalysts augmented the reaction rate. The constituent substances depicted in Figure 1 are proflavine, acriflavine, lumichrome, riboflavin tetraacetate, and lumiflavin. The current methodology facilitates the generation of 4i with diversified rates of yield. The aforementioned findings have provided a basis for improved operational productivity within the context of proflavine. According to the information presented in Table 1, entry 1, a reaction involving 0.2 mol% of proflavine led to a yield of 93%. Table 2 showcases markedly subpar outcomes for THF, DCM, EtOH, MeOH, CH_3_CN, toluene, EtOAc, CHCl_3_, DMSO, and conditions devoid of solvents exhibited a noteworthy improvement in productivity and expedited the process. In the current study, the reaction within the H_2_O context was found to demonstrate a markedly elevated rate and consequential yield. The yield of 93% was attained as per the statistical information furnished in Table 2, with specific reference to entry 3. An assortment of light sources has been utilized in research endeavors pertaining to the evaluation of the effects of blue light on crop productivity, as evidenced in Table 2. During the assessment conducted in the absence of an illuminating apparatus, the existence of 4i was observed in a minuscule quantity. The current study provides evidence that the simultaneous presence of proflavine (PFH^+^) and visible light is a crucial requirement for achieving the successful synthesis of product 4i. To establish the most favorable setups, various grades of blue-light-emitting-diode (LED) intensities, namely, 10 W, 12 W, 18 W, and 24 W were utilized. This study’s results elucidate that the utilization of 18 W blue-light-emitting-diodes (LEDs) achieved the most advantageous outcomes. Several substrates were subjected to experiments under idealized conditions, as illustrated in Table 3 and Scheme 1. The incorporation of a benzaldehyde moiety was found to not significantly influence the ultimate reaction outcome. In the current chemical reaction, the replacement of the halide functional group was deemed to be an acceptable course of action. The present state of the reaction accommodates both reactions that entail functional groups possessing the ability to donate electrons and those that entail functional groups that display electron withdrawal properties. The potential productivity of *ortho*-, *meta*-, and *para*-substituted aromatic aldehydes is markedly heightened in the natural setting. The similarity in reactivity between barbituric acid and 1,3-dimethylbarbituric acid was observed.

**TABLE 1:** The present work provides a table of optimized photocatalysts for the synthesis of **4i** ^a^.

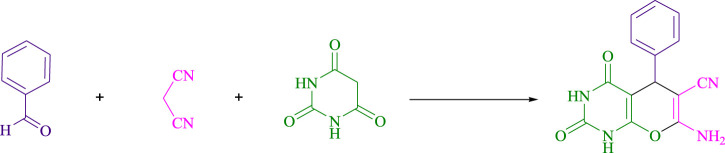				
Entry	Photocatalyst	Solvent (3 mL)	Time (min)	Isolated yields (%)
1	Proflavine (0.2 mol%)	H_2_O	3	93
2	Acriflavine (0.2 mol%)	H_2_O	3	81
3	Lumichrome (0.2 mol%)	H_2_O	3	77
4	Riboflavin tetraacetate (0.2 mol%)	H_2_O	3	75
5	Lumiflavin (0.2 mol%)	H_2_O	3	74
6	Proflavine (0.1 mol%)	H_2_O	3	78
7	Proflavine (0.3 mol%)	H_2_O	3	93
8	—	H_2_O	15	36

^a^ Reaction conditions: at ambient conditions, a total of various photocatalytic agents were coalesced with a specific quantity of 1 mmol for each of barbituric acid, benzaldehyde, and malononitrile.

**TABLE 2:** The present study aims to investigate the optimization table of solvent and visible light conditions for the synthesis of **4i** ^a^.

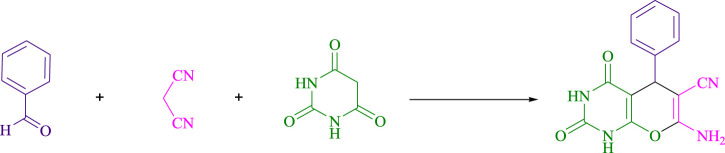				
Entry	Light source	Solvent (3 mL)	Time (min)	Isolated yields (%)
1	Blue light (10 W)	H_2_O	3	81
2	Blue light (12 W)	H_2_O	3	85
3	Blue light (18 W)	H_2_O	3	93
4	—	H_2_O	15	trace
5	White light (18 W)	H_2_O	3	76
6	Green light (18 W)	H_2_O	3	84
7	Blue light (18 W)	THF	20	15
8	Blue light (18 W)	DCM	25	13
9	Blue light (18 W)	EtOH	3	81
10	Blue light (18 W)	MeOH	4	65
11	Blue light (18 W)	—	6	42
12	Blue light (18 W)	CH_3_CN	3	68
13	Blue light (18 W)	toluene	25	19
14	Blue light (18 W)	EtOAc	3	71
15	Blue light (18 W)	CHCl_3_	25	16
16	Blue light (18 W)	DMSO	15	23
17	Blue light (24 W)	H_2_O	3	93

^a^ Reaction conditions: barbituric acid, benzaldehyde, and malononitrile were introduced to proflavine in equimolar quantities of 1 mmol each, accompanied by a photocatalyst amount of 0.2 mol%.

**TABLE 3:** The synthesis of pyrano [2,3-*d*]pyrimidine scaffolds is accomplished via the utilization of the photosensitizer (PS) biocatalyst; proflavine (PFH^+^).

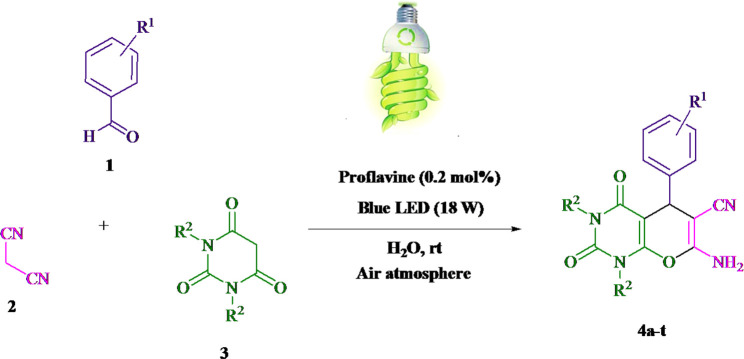			
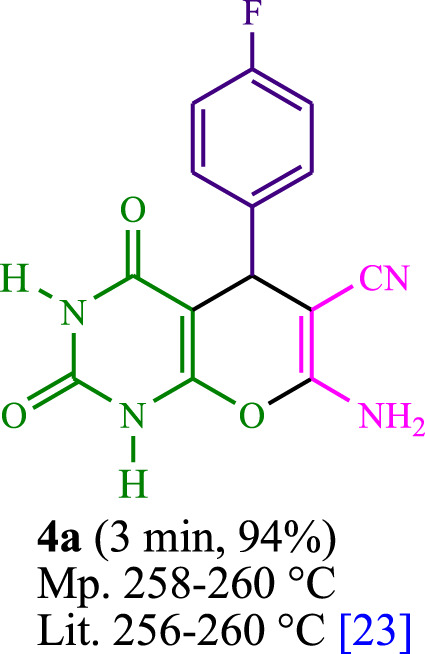 Seyyedi et al. (2016)	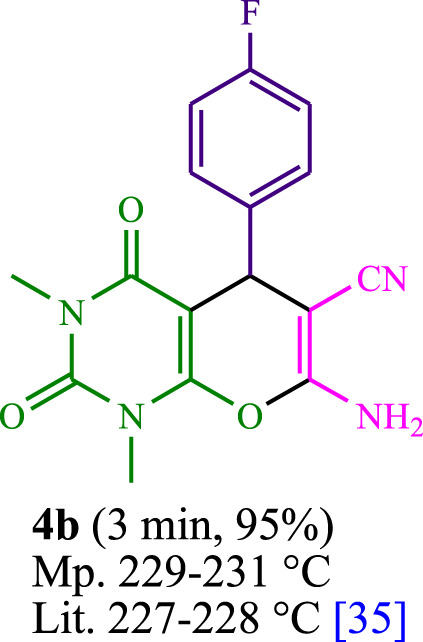 Khazaei et al. (2015b)	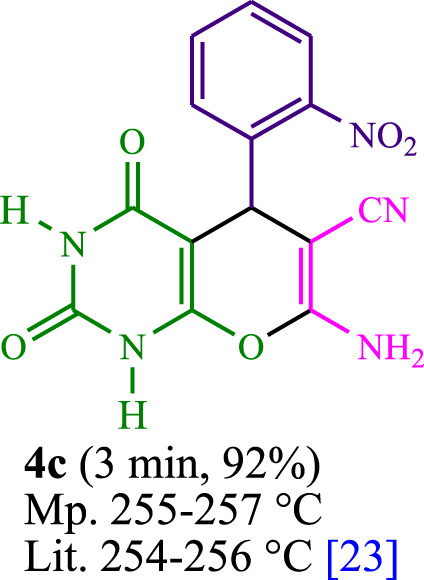 Seyyedi et al. (2016)	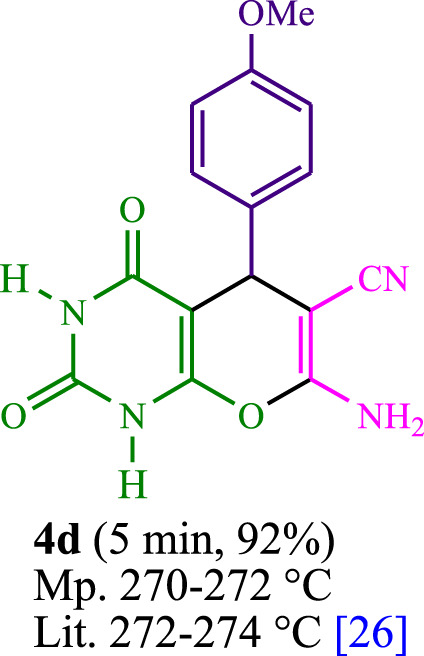 Sadeghi et al. (2015)
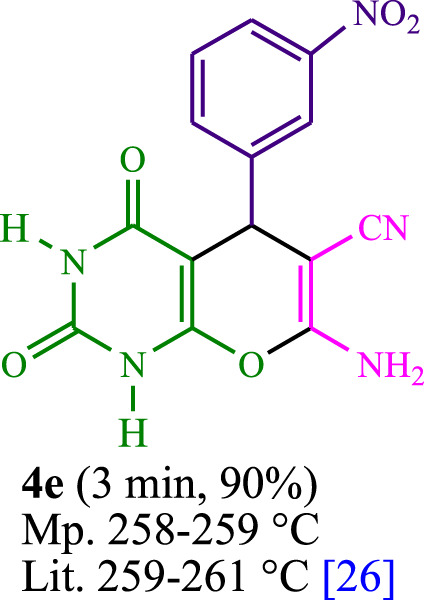 Sadeghi et al. (2015)	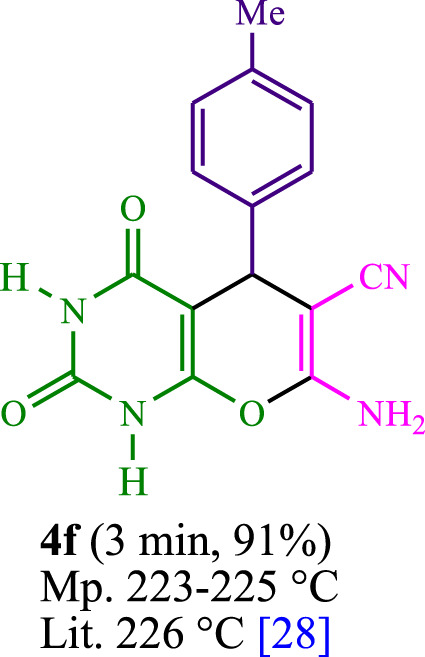 Khazaei et al. (2015a)	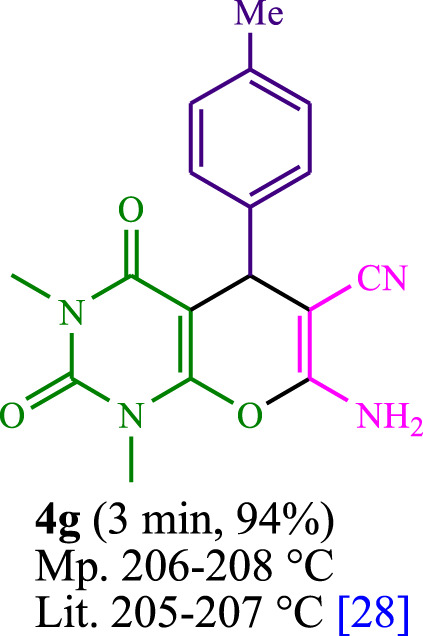 Khazaei et al., 2015a	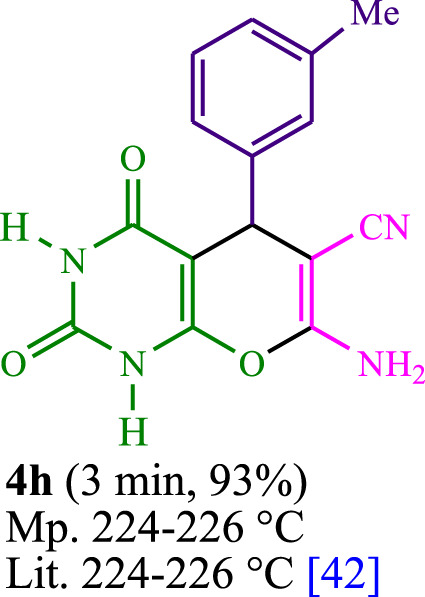 Albadi et al. (2015)
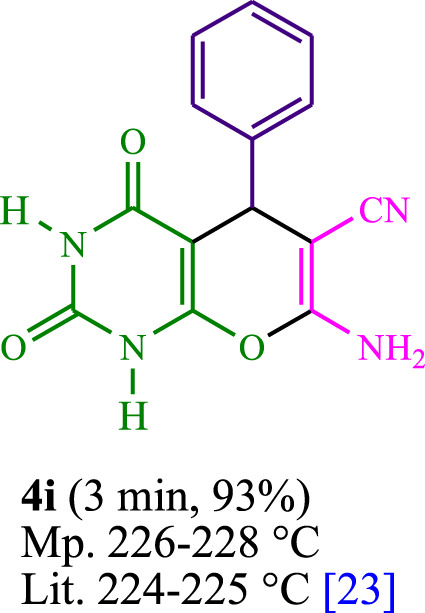 Seyyedi et al. (2016)	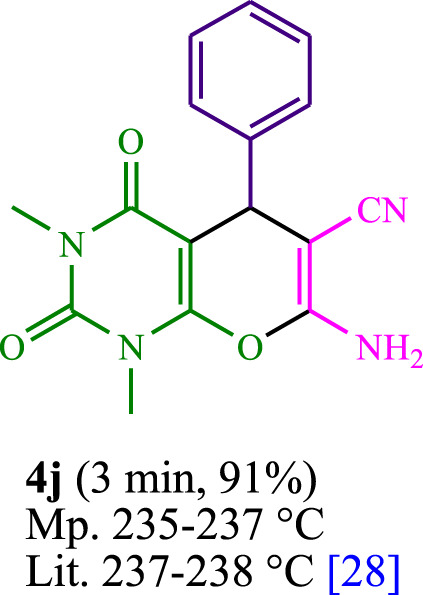 Khazaei et al. (2015a)	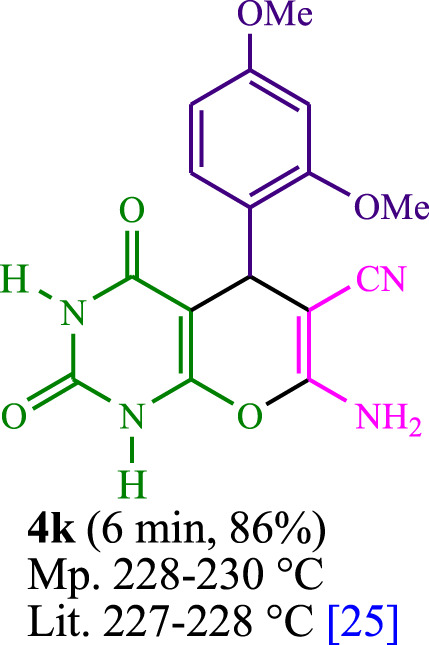 Sheihhosseini et al. (2016)	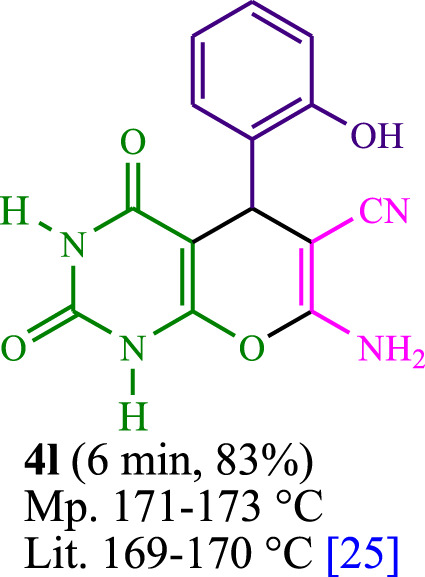 Sheihhosseini et al. (2016)
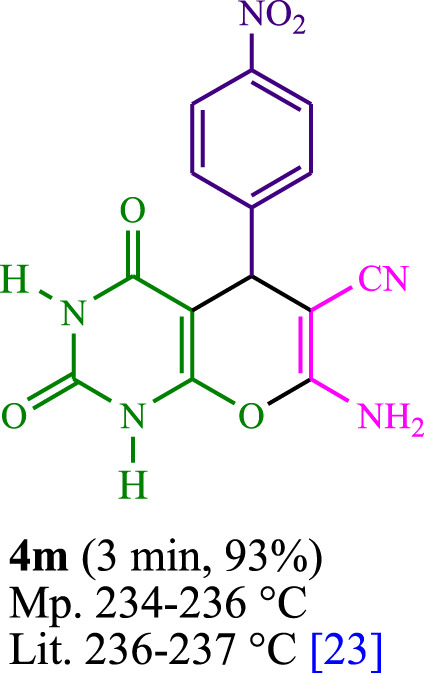 Seyyedi et al. (2016)	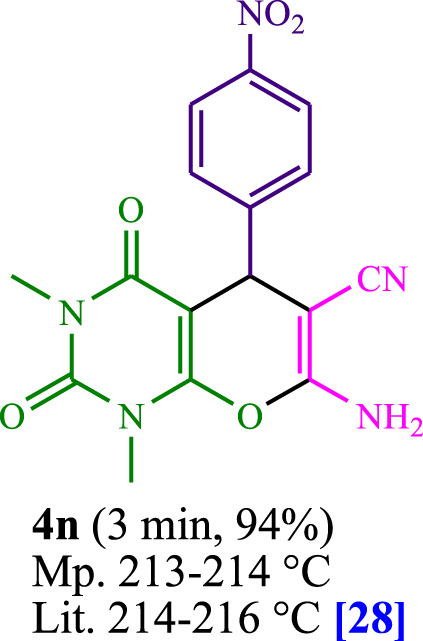 Khazaei et al. (2015a)	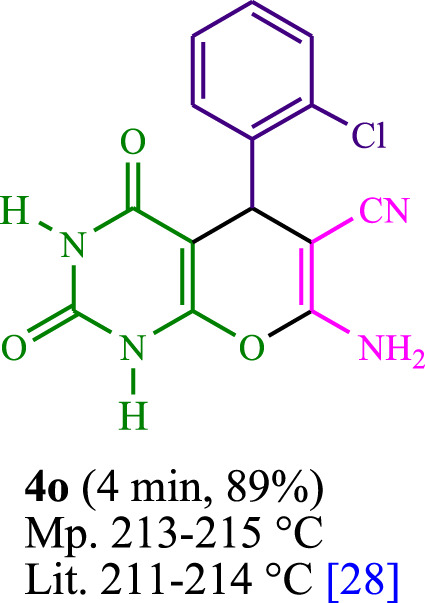 Khazaei et al. (2015a)	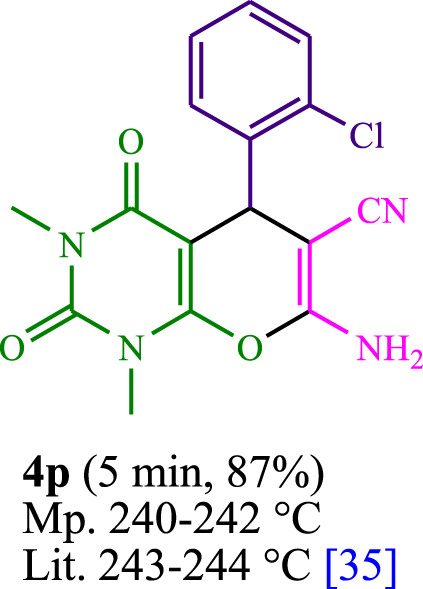 Khazaei et al. (2015b)
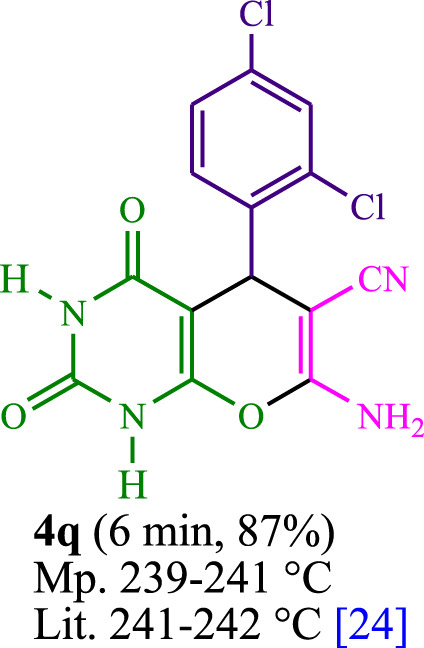 Bararjanian et al. (2009)	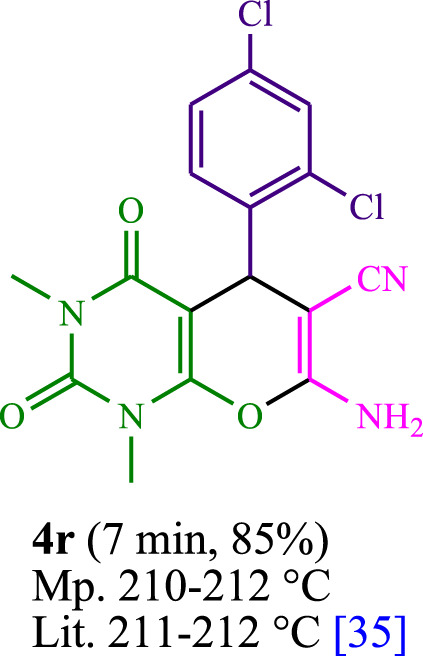 Khazaei et al. (2015b)	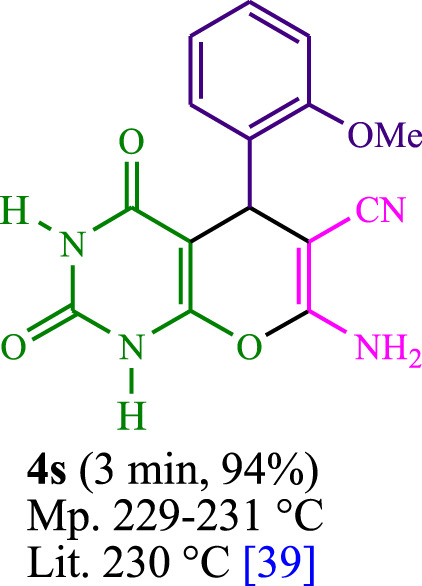 Heravi et al. (2010)	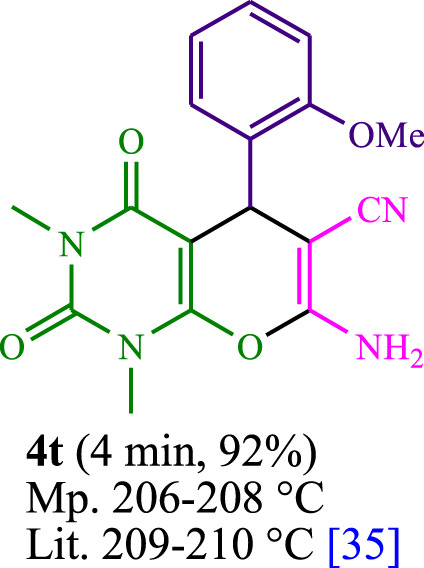 Khazaei et al. (2015b)

The presented data in Table 4 conveys definitive measures of both turnover frequency (TOF) and turnover number (TON). In academic writing, the distinct types of yield, namely the Yield/Amount of catalyst (mol) and the Yield/Time/Amount of catalyst (mol), are commonly denoted as TON and TOF, respectively. Elevated turnover number (TON) and turnover frequency (TOF) values possess the potential to augment the efficiency of a catalyst system by decreasing the quantity of catalyst necessary to optimize desired yields. Concerning 4i, a TOF measuring 155 is deemed to be elevated, whereas a TON measuring 465 is also considered to be elevated.

**TABLE 4:** The aim is to determine the numerical values of turnover number (TON) and turnover frequency (TOF) through rigorous analysis and evaluation.

Entry	Product	TON	TOF	Entry	Product	TON	TOF
1	4a	470	156.6	11	4k	430	71.6
2	4b	475	158.3	12	4l	415	69.1
3	4c	460	153.3	13	4m	465	155
4	4d	460	92	14	4n	470	156.6
5	4e	450	150	15	4o	445	111.2
6	4f	455	151.6	16	4p	435	87
7	4g	470	156.6	17	4q	435	72.5
8	4h	465	155	18	4r	425	60.7
9	4i	465	155	19	4s	470	156.6
10	4j	455	151.6	20	4t	460	115

## The suggested mechanism

**SCHEME 2:**
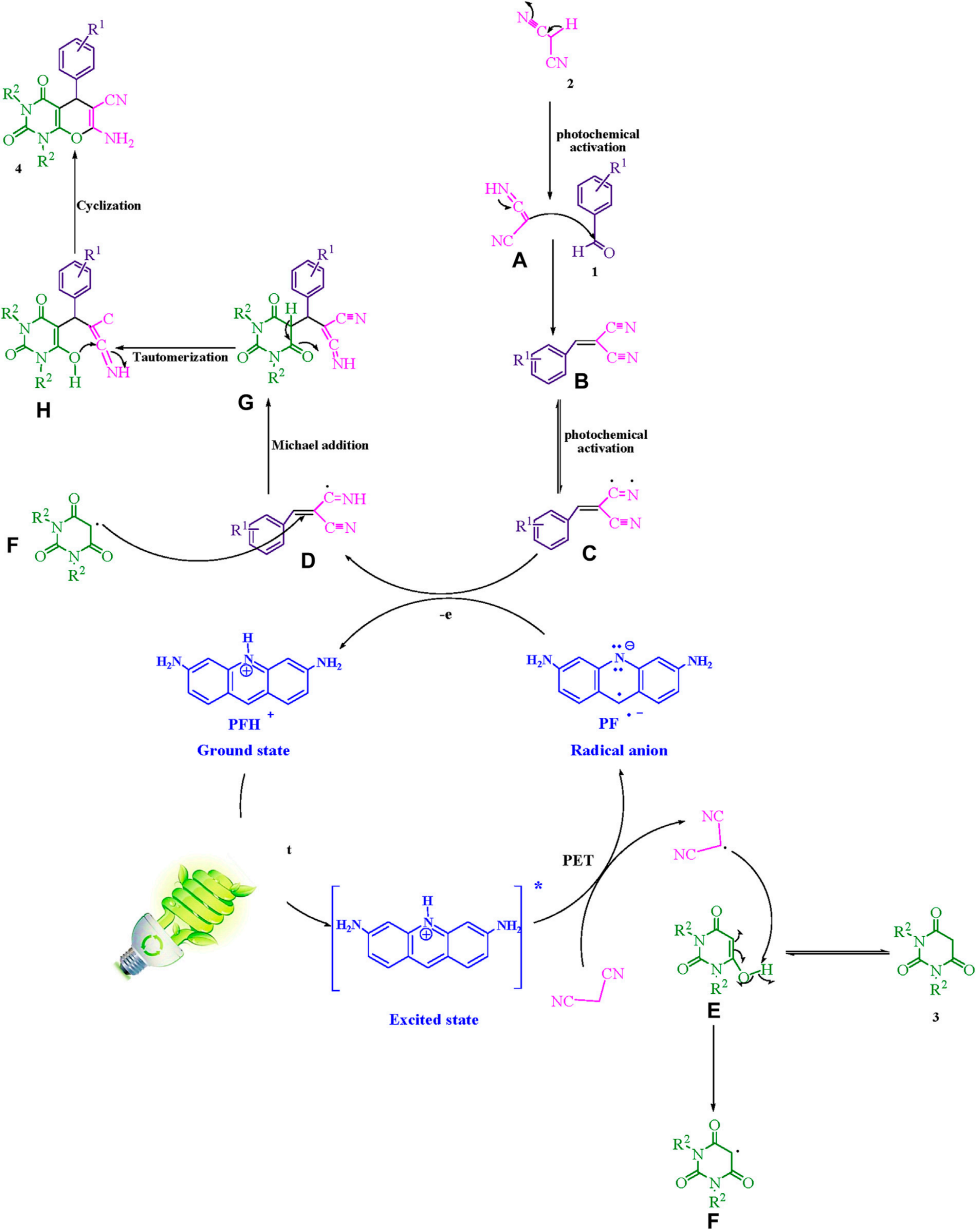
This manuscript proposes a thorough exposition of the synthetic methodology employed to produce pyrano [2,3-*d*] pyrimidine scaffolds.

Scheme 2 illustrates a comprehensive depiction of the proposed methodology. The present study employs a photoinduced-electron transfer (PET) pathway in conjunction with the bio-photocatalyst; proflavine (PFH^+^), to create photocatalytic instruments capable of utilizing visible light energy in a sustainable manner. The implementation of visible light expedites the procedural course. The generation of the malononitrile radical is facilitated by a method that originates from the mechanism of photoinduced-electron transfer (PET), thereby enhancing the performance of the [PFH^+^]^*^ and activated by visible light. The electron transfer process initiated by the radical adduct (C) and the radical anion of proflavine results in the formation of intermediates (D) as well as the ground state PFH^+^. The intermediate (F) is generated through a process of hydrogen atom abstraction from intermediate (E) by the malononitrile radical. This mechanism involves the formation of an intermediate (F) which is essential in the overall process. The intermediate species designated as (F) and (D) engage in a Michael acceptor reaction, resulting in the production of (G). Subsequently, the generation of (4) is induced by intramolecular cyclization and tautomerization mechanisms.

Table 5 provides a comparative evaluation of the efficiency of different catalysts in assisting the synthesis of pyrano [2,3-*d*] pyrimidines. The proposed methodology utilizes minuscule quantities of photocatalyst, facilitating rapid chemical conversions, while avoiding the generation of residual products. The aforementioned modality demonstrates the potential for utilization in circumstances where wavelengths of light can be feasibly observed. Atom-economy demonstrates significant efficacy and exerts considerable impact on the industrial sector at the scale of multigrams.

**TABLE 5:** The research at hand seeks to scrutinize the catalytic efficacy of various catalysts employed in the production of **4i** ^a^.

Entry	Catalyst	Conditions	Time/Yield (%)	References
1	[DABCO](SO_3_H)_2_(Cl)_2_	H_2_O, Reflux	10 min/86	Seyyedi et al. (2016)
2	[DABCO](SO_3_H)_2_(HSO_2_)_2_	H_2_O, 90°C	7 min/90	Seyyedi et al. (2016)
3	iron ore pellet	EtOH/H_2_O, Reflux	8 min/73	Sheihhosseini et al. (2016)
4	nano-sawdust-OSO_3_H	EtOH, Reflux	15 min/94	Sadeghi et al. (2015)
5	Al-HMS-20	EtOH, rt	12 h/92	Sabour et al. (2015)
6	TSA	EtOH/H_2_O, Reflux	90 min/88	Khazaei et al. (2015a)
7	B(OH)_3_	THF/H_2_O, Reflux	125 min/81	Khazaei et al. (2015a)
8	cellulose-based nanocomposite	THF/H_2_O, Reflux	35 min/90	Maleki et al. (2017)
9	DBA	EtOH/H_2_O, Reflux	58 min/94	Bhat et al. (2016)
10	proflavine (PFH^+^)	Blue LED, H_2_O, rt	3 min/93	This work

^a^ The synthesis that is proposed involves the application of three components, namely benzaldehyde, malononitrile, and barbituric acid.

## Conclusion

The Knoevenagel-Michael cyclocondensation reaction, which is initiated by radicals, has been applied in the green synthesis of pyrano [2,3-*d*] pyrimidine frameworks via the reaction of aryl aldehydes, malononitrile, and either barbituric acid or 1,3-dimethylbarbituric acid. The current investigation employed a novel photosensitizer (PS) biocatalyst; proflavine (PFH^+^), as an organo-photocatalyst via a photoinduced-electron transfer (PET). At ambient temperature and under air atmosphere conditions, the efficacy of blue-light-emitting-diode (LED) technology has been found to generate a sustainable mechanism of energy production in an aqueous environment. The method presented in this study confers several advantages to the domain of chemical synthesis. The advantages of this approach pertain to several aspects, including rapid reaction times, elimination of hazardous solvents, increased product yields, optimized reaction pathways, robust operating conditions, and utilization of sustainable energy sources. The employment of chromatography was not deemed indispensable for the process of separation. By safeguarding the outcome, it is possible to expedite a multigram-scale reaction involving various substrates. Henceforth, the proposed methodology can be effectively executed within a framework that adheres to the principles of sustaining long-term ecological and financial sustenance.

## Data Availability

The original contributions presented in the study are included in the article/Supplementary Material, further inquiries can be directed to the corresponding authors.
