# Supplementary material for: Proflavine (PFH+): as a photosensitizer (PS) biocatalyst for the visible-light-induced synthesis of pyrano [2,3-d] pyrimidine scaffolds
Source: Front Chem. 2024 Mar 26;12:1304850. doi: 10.3389/fchem.2024.1304850 (PMC11002269; doi:10.3389/fchem.2024.1304850)
Supplement: Supplementary file 1 [file DataSheet1.PDF]

Supporting Information

Proflavine (PFH^+): as a photosensitizer (PS) biocatalyst for the visible-light-induced synthesis of pyrano[2,3-*d*]pyrimidine scaffolds

Farzaneh Mohamadpour[a,*], Ali Mohammad Amani[a,*]

[a]Department of Medical Nanotechnology, School of Advanced Medical Sciences and Technologies, Shiraz University of Medical Sciences, Shiraz, Iran

Corresponding authors email: f_mohamadpour@sums.ac.ir; mohamadpour.f.7@gmail.com; amani_a@sums.ac.ir, aliamani@sums.ac.ir

7-amino-5-(2-nitrophenyl)-2,4-dioxo-2,3,4,5-tetrahydro-1H-pyrano[2,3-d]pyrimidine-6-carbonitrile (4c)

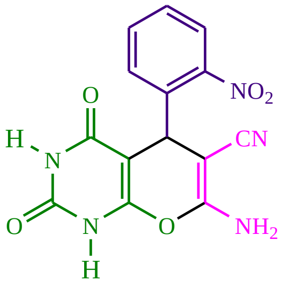

Yield: 92%; m.p. 255-257 °C; ^{1}HNMR (300 MHz, DMSO-d_6): 4.27 (1H, s, CHAr), 7.69-8.24 (6H, m, ArH & NH_2), 10.51 (1H, s, NH), 11.64 (1H, s, NH).

7-Amino-5-(2,4-dimethoxyphenyl)-2,4-dioxo-2,3,4,5-tetrahydro-1H-pyrano[2,3-d]-pyrimidine-6-carbonitrile (4k)

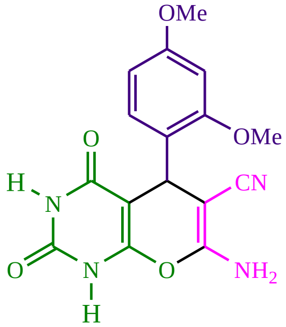

Yield: 86%; m.p. 228-230 °C; ^{1}HNMR (300 MHz, DMSO-d_6): 3.71 (3H, s, OCH_3), 3.76 (3H, s, OCH_3), 4.56 (1H, s, CHAr), 7.13-7.53 (5H, m, ArH & NH_2), 10.04 (1H, s, NH), 11.29 (1H, s, NH).

7-Amino-5-(2,4-dichloroyphenyl)-2,4-dioxo-2,3,4,5-tetrahydro-1H-pyrano[2,3-d]-pyrimidine-6-carbonitrile (4q)

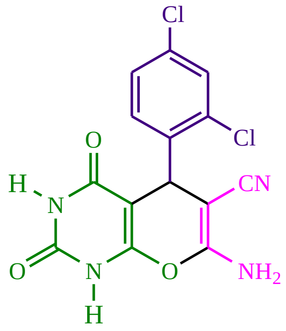

Yield: 87%; m.p. 239-241 °C; ^{1}HNMR (300 MHz, DMSO-d_6): 4.23 (1H, s, CHAr), 7.57-8.11 (5H, m, ArH & NH_2), 10.68 (1H, s, NH), 11.73 (1H, s, NH).

7-Amino-5-(2-methoxyphenyl)-2,4-dioxo-2,3,4,5-tetrahydro-1H-pyrano[2,3-d]-pyrimidine-6-carbonitrile (4s)

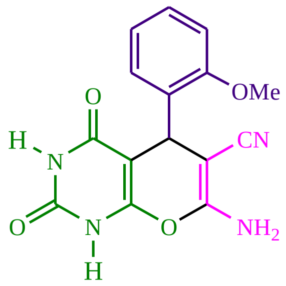

Yield: 94%; m.p. 229-231 °C; ^{1}HNMR (300 MHz, DMSO-d_6): 3.79 (3H, s, OCH_3), 4.49 (1H, s, CHAr), 7.46-7.78 (4H, m, ArH & NH_2), 8.18 (2H, t, J = 8.0 Hz, ArH), 10.58 (1H, s, NH), 11.36 (1H, s, NH).
